# Factors associated with depression, anxiety, stress, PTSD, and fatigue of medical staff during the COVID-19 pandemic in Shanghai: a two-phase cross-sectional study

**DOI:** 10.1590/1414-431X2024e13943

**Published:** 2025-03-03

**Authors:** Yunyue Li, Xing Wang, Minghui Li, Bo Hu, Junlai Cheng, Hongguang Chen, Xiaotong Li, Shihan Zhu, Mengqian Li

**Affiliations:** 1 Department of Psychosomatic Medicine, The 1st Affiliated Hospital, Jiangxi Medical College, Nanchang University, Nanchang, Jiangxi, China; 2 Clinical Medicine Center, Jiangxi Medical College, Nanchang University, Nanchang, Jiangxi, China; 3 Tianjin Anding Hospital, Mental Health Center of Tianjin Medical University, Tianjin, China; 4 Renji Hospital, Shanghai Jiao Tong University School of Medicine, Shanghai, China; 5 Peking University Sixth Hospital, Peking University Institute of Mental Health, NHC Key Laboratory of Mental Health (Peking University), National Clinical Research Center for Mental Disorders (Peking University Sixth Hospital), Beijing, China; 6 Queen Mary School, Jiangxi Medical College, Nanchang University, Nanchang, Jiangxi, China; 7 Department of Psychology, School of Public Policy and Management, Nanchang University, Nanchang, Jiangxi, China

**Keywords:** COVID-19 in Shanghai, Depression, Anxiety, Stress, PTSD, Fatigue

## Abstract

During the COVID-19 pandemic in Shanghai, medical workers were more vulnerable to psychological problems. This two-phase cross-sectional survey was conducted by online questionnaires to investigate the symptoms of depression, anxiety, stress, post-traumatic stress disorder (PTSD), and fatigue in healthcare workers during the outbreak of COVID-19 and after the resumption of work and production in Shanghai. The questionnaire included the Depression Anxiety Stress Scale-21 (DASS-21), the Impact of Event Scale-Revised (IES-R), and the Fatigue Assessment Instrument (FAI). In Phase I (n=2192), the prevalence of depression, anxiety, stress, and PTSD symptoms among medical staff was 45.48, 41.93, 20.35, and 75.55%. In Phase II (n=1031), after work resumed in Shanghai, the prevalence was 19.79, 21.44, 28.23, and 12.22%, respectively. Fatigue had a mean score of 121.23±45.776 in Phase I and 144.73±44.141 in Phase II. Binary logistic regression identified risk factors associated with this psychological status: personal and familial chronic disease history; occupation, including doctor, nurse, or administrative staff; working in the fever clinic, infectious disease department, emergency or intensive care unit, hemodialysis room, or clinical laboratory; work experience of 3-6 years or 7-10 years; and involvement in nucleic acid sampling team. Medical staff self-reported comparatively high rates of depression, anxiety, stress, and, especially, PTSD symptoms during the COVID-19 pandemic in Shanghai. Our study indicated that after work resumption in Shanghai, it appeared that the overall mental health of medical staff improved somewhat. Nevertheless, the high level of fatigue exhibited still cannot be ignored.

## Introduction

Public health emergencies have a profound negative impact on healthcare workers' mental well-being. The stress and psychological burden on healthcare personnel dealing with these situations are heightened due to the need to care for contagious patients while ensuring their own safety. Uncertainty in disease dynamics and limited resources exacerbate feelings of anxiety, depression, emotional exhaustion, and post-traumatic stress symptoms (1). Such challenges are often exemplified by infectious disease outbreaks.

Since the first report of the COVID-19 virus, new cases were reported worldwide and countries struggled during that period. Latin America and the Caribbean (LAC), for example, were the hardest hit by the COVID-19 pandemic and are home to eight of the 10 countries with the highest mortality rates in the world (2). Since then, there has been a sporadic distribution of cases in Shanghai, China, which has remained within manageable limits. Nevertheless, the new variant strain of the novel coronavirus, Omicron, ravaged Shanghai starting in March 2022. As of April 13, 2022 more than 46,000 cases had been confirmed in Shanghai, with more than 200,000 asymptomatic patients (3). Genetic sequencing of the virus revealed that the Omicron variant (Omicron BA.2) was the predominant strain in the current outbreak (4). It was found to be more transmissible and infectious, with higher drug resistance and pathogenicity than previous variants (5).

In early 2021, Shanghai began widespread COVID-19 vaccination, particularly with the rollout of booster shots. Despite this, the increase in asymptomatic infections and significant community transmission indicated that while vaccines played a crucial role in reducing severe cases, controlling the outbreak became increasingly challenging. Additionally, the overlap with routine patient care exceeded available medical resources (6), placing healthcare workers at greater risk and negatively impacting their well-being. As the backbone of emergency public health responses, healthcare staff faced not only the anxiety of managing a high volume of COVID-19 patients but also the fear of close contact and potential infection. The heavy workload and psychological pressure predisposed them to various degrees of anxiety, depression, and panic attacks (7).

Medical professionals are affected by depression, anxiety, and post-traumatic stress disorder (PTSD). Numerous investigations conducted in Wuhan during the beginning of the COVID-19 outbreak revealed adverse emotions among medical staff. In this regard, depression and anxiety symptoms are among the most prevalent psychiatric disorders, with the detection rate among medical staff exceeding 40% at that time (8,9). The large patient volume, widespread disease, high mortality rate, and the regular exposure of medical staff to trauma and death all contribute to the development of PTSD symptoms (10). In addition, medical staff suffering from PTSD symptoms constantly repeated the experience of working on the frontline of the COVID-19 epidemic. These symptoms, often accompanied by intense fear and physical sensations, may endure for at least a few weeks and severely impact the work, life, and other elements of their daily existence (11). Meanwhile, due to the nature of their work, medical staff frequently contact COVID-19 patients. The threat of infection instills fear among them even with personal protective equipment. Especially in the early stages of an epidemic, when little was known about the novel virus, there was significant pressure on medical staff due to the lack of effective treatments or medications available to combat the illness (12). Studies indicated that several factors, including the severity of the epidemic, family support, social support, and personal job intensity, might affect the stress experienced by medical staff during the COVID-19 epidemic (13). Moreover, they may experience physical and psychological exhaustion during a protracted battle against the COVID-19 epidemic due to intense labor, which can further negatively affect their psychological well-being (14). They experience extreme tiredness from their work as a result of excessive fatigue and high levels of stress, or they may even develop psychosomatic exhaustion syndrome (15), which can cause negative psychological reactions including depression, anxiety, and irritability.

Most research has concentrated on the mental health of the medical staff at the early stage of the outbreak. Nevertheless, the psychological state of the medical staff could vary at various points and stages of the outbreak. This is especially relevant given that the Omicron variant is more contagious and transmissible, potentially consuming more medical resources. As an occupational group in close contact with COVID-19 patients, the psychological state of medical staff in Shanghai deserves attention. This research aimed to analyze the risk variables connected to depression, anxiety, stress, fatigue, and PTSD symptoms among the medical staff in Shanghai following the current outbreak. Four months after the initial survey, after the current pandemic ended and work and production had resumed, we investigated the prevalence of these symptoms among medical staff once again. The extensive effects of the pandemic on the mental health of medical staff were explored through this two-phase cross-sectional survey. Our hypothesis was that during the COVID-19 outbreak, the medical staff suffered from elevated levels of anxiety, depression, stress, fatigue, and PTSD symptoms. We anticipated that there was a significant correlation between the psychological symptoms of the medical staff and factors connected to their work. With the full resumption of work in Shanghai, we then hypothesized that the percentage of medical professionals experiencing psychological symptoms would be comparatively low.

## Material and Methods

### Sample

We carried out a two-phase cross-sectional study to investigate the mental health of medical staff in Shanghai, China. Our study included doctors, nurses, medical technicians, and administrative staff who work in the hospital. Subjects who had encountered significant changes in their families or personal lives that were unrelated to the COVID-19 pandemic during the study period, as well as those with a history of psychiatric illnesses, were excluded from the investigation. The Phase I survey was conducted from April 17, 2022, to May 10, 2022, during the peak of COVID-19 in Shanghai. The Phase II survey was performed after the full resumption of work and production in Shanghai, covering the period from 8 August 2022 to 23 September 2022. Online questionnaires were used to collect data from the medical staff working in the Renji Hospital of Shanghai Jiaotong University Hospitals, Shanghai Xinhua Hospital, Shanghai Sixth People's Hospital, Shanghai Children's Medical Center, and medical teams assisting Shanghai. The questionnaire took 8-15 min to complete. We removed questionnaires with missing items and too short or too long response times from all subjects. In the first phase, 2217 participants were investigated, which contained 2192 valid questionnaires with a final response rate of 98.87%. In the second stage of the survey, 1056 participants were investigated, and 1031 valid questionnaires were included, giving a final response rate of 97.63%.

We investigated sociodemographic characteristics with a self-designed questionnaire. The Depression Anxiety Stress Scale (DASS-21), Impact of Event Scale-Revised (IES-R), and Fatigue Assessment Instrument (FAI) were also utilized in this study to assess the psychological symptoms of participants. Everyone was aware of the procedure, scope, and objectives of the survey before the questionnaires were distributed. Each participant filled out the online survey voluntarily and anonymously. The study was approved by the Research Ethics Board of the First Affiliated Hospital of Nanchang University (approval number: 2020-048).

### Socio-demographic characteristics

We investigated the participants' gender, age, educational level, marital status, history of chronic disease, occupation, department of work, hospital type, technical title, working years, average working hours per day during the COVID-19 epidemic, workplace and living place during the COVID-19 epidemic in Shanghai, and history of chronic disease in family members.

### DASS-21

The DASS-21 is a self-report scale used to assess three psychological aspects including depression, anxiety, and stress in individuals. This study used the 21-item revision to the DASS scale, which originally had 42 items. The depression, anxiety, and stress subscales are all included in the scale, scored on a 4-point scale with 0 representing “did not apply to me at all” and 3 representing “applied to me very much or most of the time”. The final score of each subscale was calculated by multiplying by 2 each scale's score (16). The DASS-21 has strong reliability and validity and can be used to assess the mental status of medical staff (17). In our research, the reliability analysis of the scale revealed that Cronbach's α was 0.959 for the first survey and 0.987 for the second survey after 4 months.

### IES-R

The IES-R is a self-report scale used to explore an individual's distress in response to a specific life event. The 22-item scale consists of three subscales: avoidance, intrusion, and hyperarousal. This 5-point Likert-type scale scores range from 0 to 4 on a scale from “not at all” to “extremely” with a possible total score of 0 to 88. The subscale scores were added to obtain the individual's total score (18). The scale had strong reliability, with internal consistency coefficients of 0.89 to 0.96 for the total scale and the three factors. The Cronbach's α of the scale in our study was 0.966 at the time of the outbreak and 0.979 four months later.

### FAI

The FAI was used to evaluate the fatigue level of subjects over the last two weeks. This 29-item scale is rated on a 7-point Linkert scale, with each item responded on a scale from “completely disagree” to “completely agree”. Higher scores indicate more severe fatigue. In addition, the scale is composed of four factors, which assess the fatigue characteristics of patients from four different aspects. Factor 1 (S) is the fatigue severity scale, which measures the degree of fatigue; factor 2 (SS) is the fatigue environment specificity scale, which measures the sensitivity of fatigue to specific environments and assesses whether fatigue is context-specific; factor 3 (PC) is the fatigue outcome scale, which measures the possible psychological consequences of fatigue; factor 4 (RTR/S) is the response to rest and sleep scale, which measures whether fatigue is responsive to rest or sleep (19). At the time of the epidemic outbreak and four months later, Cronbach's α of this scale was 0.973 and 0.978, respectively.

### Statistics

Differences in sociodemographic, work-related, and family-related variables were compared between subgroups with and without depression, anxiety, stress, and PTSD symptoms using chi-squared tests. Differences between groups in levels of fatigue were examined using independent samples *t*-tests and one-way analysis of variance (ANOVA). Finally, binary logistic regression was applied to assess the independent associations between demographic characteristics and work-related factors and depression, anxiety, stress, and PTSD symptoms among medical staff; multiple linear regression was utilized to evaluate the factors affecting the fatigue status of medical staff.

## Results

### Socio-demographic information

Among all participants in the Phase I survey, 1590 (72.5%) were female. Most participants (68.8%) were aged between 30 to 50 years. Nearly half of the participants (49.2%) had a bachelor's degree, while 19.4% obtained a master's degree or more. The majority of participants (71.4%) were married. Among all participants, 18.6% had a history of chronic disease, and 47.1% of participants' family members had a history of chronic disease.

Among all subjects in the Phase II survey, 48.8% were female. The majority of participants (79.1%) were in the range of 30 to 50 years of age. Most of the participants (67.2%) had a bachelor's degree, while 6.7% obtained a master's degree or more. An overwhelming majority of participants (81.0%) were married. Among all participants, 70.1% had a history of chronic disease, and 76.8% of participants' family members had a history of chronic disease. In addition, data regarding the medical staff's employment was acquired (Supplementary Table S1).

### Prevalence of depression, anxiety, stress, and PTSD symptoms

Supplementary Table S2 displays the distribution of the medical staff presenting with symptoms of depression, anxiety, stress, and PTSD across different sociodemographic characteristics in the Phase I survey, with the prevalence of symptoms being 45.48, 41.93, 20.35, and 75.55%, respectively. The chi-squared test results revealed significant differences in the likelihood of depression, anxiety, stress, and PTSD symptoms by age range, educational level, occupation, department of work, technical title, working years, average working hours per day since the epidemic, chronic disease history in participants, and chronic disease history in family members (Supplementary Table S2).

The prevalence of depression, anxiety, stress, and PTSD symptoms among the medical staff after the full resumption of work and production in Shanghai was 19.79, 21.44, 28.23, and 12.22%, respectively (Supplementary Table S3). The chi-squared test results revealed significant differences in the prevalence of depression, anxiety, stress, and PTSD symptoms by gender, age range, marital status, chronic disease history in participants and family members, occupation, department of work, technical title, working years, average working hours per day, workplace, and living place since Shanghai lifted lockdown (Supplementary Table S3).

### Factors associated with depression and anxiety symptoms

Factors associated with self-reported depression symptoms in the Phase I survey included: history of chronic disease, occupation, time in job, and average working hours per day since COVID-19 epidemic, workplace during COVID-19 epidemic in Shanghai, designated hospital, and family member with a history of chronic disease (Table 1). In the Phase II survey, associated factors were chronic disease, party affairs medical management department, logistics support department, junior technical title, and average working hours since end of lockdown less than 9 h (Table 2).

**Table 1:** Factors associated with depression, anxiety, stress, and post-traumatic stress disorder (PTSD) among medical staff in the Phase I survey.

Characteristics	DepressionOR (95%CI)	AnxietyOR (95%CI)	StressOR (95%CI)	PTSDOR (95%CI)
Gender (Reference: Female)				
Male	1.09 (0.87-1.38)	1.03 (0.81-1.30)	1.10 (0.84-1.46)	0.83 (0.65-1.07)
Age (Reference: >50 years)				
<30 years	1.02 (0.63-1.64)	1.50 (0.89-2.40)	1.19 (0.65-2.19)	1.22 (0.75-2.00)
30-50 years	1.26 (0.84-1.88)	1.56 (1.03-2.38)*	1.18 (0.70-2.00)	1.33 (0.89-2.00)
Educational level (Reference: Postgraduate or more)				
Junior college or less	1.01 (0.72-1.40)	1.18 (0.83-1.66)	0.73 (0.49-1.10)	0.67 (0.47-0.98)*
Undergraduate	0.99 (0.74-1.32)	1.31 (0.97-1.77)	0.80 (0.56-1.12)	0.86 (0.62-1.20)
Chronic disease (Reference: No)				
Yes	1.88 (1.46-2.41)***	2.26 (1.75-2.92)***	1.47 (1.10-1.95)**	1.69 (1.24-2.31)**
Occupation (Reference: Others)				
Doctor	2.17 (1.33-3.54)**	1.28 (0.76-2.15)	1.73 (0.90-3.30)	1.13 (0.67-1.89)
Nurse	3.03 (1.92-4.76)***	2.08 (1.30-3.36)**	2.48 (1.35-4.54)**	1.49 (0.94-2.36)
Medical technicians	1.30 (0.79-2.12)	0.92 (0.54-1.57)	1.51 (0.78-2.92)	0.95 (0.58-1.56)
Administrative staff	3.39 (1.77-6.49)***	2.54 (1.31-4.92)**	3.31 (1.52-7.20)**	0.90 (0.46-1.76)
Department of work (Reference: Others)				
Fever clinic/Infectious disease department	0.55 (0.28-1.08)	0.49 (0.24-0.99)*	0.67 (0.30-1.52)	0.72 (0.36-1.47)
Emergency/Intensive Care Unit/Hemodialysis Room	1.08 (0.69-1.68)	1.48 (0.94-2.33)	0.80 (0.48-1.34)	1.31 (0.76-2.26)
Clinical laboratory	1.43 (0.88-2.32)	1.82 (1.10-3.00)*	1.07 (0.60-1.91)	1.53 (0.88-2.69)
Other clinical departments	0.81 (0.61-1.08)	0.93 (0.69-1.25)	0.75 (0.53-1.06)	1.10 (0.80-1.52)
Party affairs medical management department	0.56 (0.22-1.42)	0.55 (0.21-1.43)	0.80 (0.30-2.19)	1.12 (0.40-3.14)
Logistics support department	0.49 (0.28-0.86)*	0.62 (0.36-1.07)	0.37 (0.17-0.82)*	0.95 (0.57-1.58)
Technical title (Reference: Seniors)				
No	0.80 (0.50-1.27)	0.79 (0.48-1.28)	0.97 (0.55-1.71)	1.08 (0.65-1.81)
Junior	1.02 (0.69-1.52)	1.20 (0.79-1.81)	1.03 (0.65-1.65)	1.44 (0.91-2.26)
Intermediate	1.01 (0.69-1.47)	1.04 (0.70-1.55)	0.95 (0.61-1.49)	1.18 (0.76-1.82)
Working years (Reference: >10)				
<3	1.99 (1.41-2.82)***	1.73 (1.21-2.48)**	1.20 (0.77-1.86)	0.81 (0.56-1.17)
3-6	1.70 (1.26-2.29)**	1.50 (1.10-2.04)	1.29 (0.90-1.85)	0.80 (0.58-1.11)
7-10	2.08 (1.58-2.75)***	1.53 (1.16-2.03)**	1.65 (1.19-2.27)**	1.17 (0.85-1.62)
Average working hours since the epidemic (hours/day) (Reference: >12)				
<9	0.60 (0.42-0.85)**	0.51 (0.36-0.73)***	0.61 (0.40-0.92)*	0.47 (0.31-0.71)***
9-10	0.80 (0.56-1.16)	0.97 (0.67-1.40)	1.03 (0.68-1.58)	0.82 (0.52-1.28)
11-12	0.96 (0.64-1.45)	1.05 (0.70-1.59)	1.27 (0.80-2.01)	0.75 (0.46-1.22)
Present workplace (Reference: Others)				
Mobile cabin hospital	0.68 (0.49-0.93)*	1.26 (0.91-1.74)	1.23 (0.82-1.83)	0.85 (0.60-1.20)
Designated hospital	0.63 (0.45-0.89)**	1.14 (0.80-1.63)	1.20 (0.78-1.85)	0.92 (0.63-1.36)
Nucleic acid sampling team	1.70 (0.79-3.66)	2.43 (1.14-5.20)*	1.60 (0.73-3.47)	1.17 (0.48-2.83)
Stayed at the hospital	0.92 (0.71-1.19)	1.14 (0.87-1.49)	1.35 (0.97-1.88)	0.85 (0.64-1.13)
History of chronic disease in family members (Reference: No)				
Yes	1.51 (1.25-1.82)***	1.62 (1.34-1.97)***	1.54 (1.22-1.95)***	1.21 (0.97-1.50)

*P<0.05, **P<0.01, ***P<0.001 (univariate logistic regression).

**Table 2:** Factors associated with depression, anxiety, stress, and post-traumatic stress disorder (PTSD) among medical staff in the Phase II survey.

Characteristics	DepressionOR (95%CI)	AnxietyOR (95%CI)	StressOR (95%CI)	PTSDOR (95%CI)
Gender (Reference: Female)				
Male	1.19 (0.65-2.17)	1.06 (0.55-2.05)	1.00 (0.51-1.99)	1.09 (0.59-2.03)
Age (Reference: >50 years)				
<30	0.67 (0.19-2.29)	1.62 (0.40-6.53)	0.57 (0.15-2.20)	0.62 (0.17-2.18)
30-50	1.38 (0.62-3.10)	1.97 (0.83-4.67)	1.83 (0.70-4.75)	0.92 (0.42-2.03)
Educational level (Reference: Postgraduate or more)				
Junior college or less	0.77 (0.26-2.28)	2.03(0.61-6.75)	2.46 (0.67-9.04)	-
Undergraduate	0.77 (0.31-1.95)	1.96 (0.70-5.48)	3.29 (1.04-10.48)*	-
Marital status (Reference: Others)				
Married	1.09 (0.56-2.12)	1.22 (0.59-2.53)	1.94 (0.83-4.54)	1.19 (0.60-2.38)
Chronic disease (Reference: No)				
Yes	4.34 (2.36-7.97)***	4.95 (2.63-9.32)***	3.69 (1.95-6.99)***	5.99 (2.87-12.48)***
Occupation (Reference: Others)				
Doctor	2.01 (0.41-9.86)	4.30 (0.68-27.43)	5.04 (0.51-49.91)	2.67 (0.58-12.26)
Nurse	1.32 (0.32-5.40)	2.42 (0.46-12.63)	1.22 (0.15-10.20)	1.27 (0.34-4.73)
Medical technicians	1.00 (0.26-3.85)	1.73 (0.35-8.63)	0.62 (0.08-5.01)	1.69 (0.49-5.78)
Administrative staff	1.68 (0.34-8.18)	5.82 (0.99-34.45)	3.38 (0.37-31.28)	1.85 (0.45-7.59)
Department of work (Reference: Others)				
Fever clinic/Infectious disease department	2.48 (0.80-7.71)	3.17 (0.96-10.42)	6.57 (2.02-21.41)**	2.53 (0.75-8.54)
Emergency/Intensive Care Unit/Hemodialysis Room	1.77 (0.63-4.97)	2.93 (0.96-8.97)	4.84 (1.64-14.34)**	1.59 (0.53-4.75)
Clinical laboratory	3.18 (0.79-12.70)	3.77 (0.88-16.23)	11.39 (2.50-52.00)**	3.22 (0.59-17.64)
Other clinical departments	0.59 (0.29-1.23)	0.60 (0.28-1.30)	0.59 (0.24-1.48)	1.16 (0.55-2.43)
Party affairs medical management department	0.04 (0.01-0.37)**	0.08 (0.01-0.54)*	-	0.77 (0.16-3.66)
Logistics support department	0.11 (0.02-0.49)**	0.24 (0.05-1.20)	0.10 (0.01-1.05)	0.93 (0.28-3.09)
Technical title (Reference: Seniors)				
No	1.69 (0.30-9.42)	1.62 (0.26-10.20)	1.70 (0.18-15.74)	1.03 (0.22-4.80)
Junior	3.77 (1.11-12.87)*	3.59 (0.97-13.27)	3.45 (0.74-16.21)	2.05 (0.65-6.45)
Intermediate	1.91 (0.60-6.09)	2.15 (0.62-7.40)	1.90 (0.43-8.40)	1.78 (0.58-5.46)
Working years (Reference: >10)				
<3	0.99 (0.29-3.37)	0.57 (0.15-2.17)	2.14 (0.48-9.54)	1.37 (0.43-4.41)
3-6	1.55 (0.61-3.96)	1.75 (0.63-4.88)	1.83 (0.67-5.00)	1.47 (0.55-3.92)
7-10	2.01 (0.92-4.39)	2.29 (1.01-5.20)*	3.32 (1.41-7.79)**	2.30 (0.99-5.37)
Average working hours since Shanghai is fully liberated (hours/day) (Reference: >12)				
<9	0.11 (0.02-0.47)**	0.05 (0.01-0.23)***	0.60 (0.01-0.25)***	0.46 (0.11-1.87)
9-10	0.23 (0.05-1.00)	0.15 (0.03-0.75)*	0.16 (0.04-0.65)*	0.57 (0.14-2.38)
11-12	0.55 (0.11-2.71)	0.34 (0.06-1.85)	0.55 (0.12-2.45)	1.20 (0.25-5.80)
Present workplace (Reference: Others)				
Designated hospital	1.70 (0.65-4.45)	1.51 (0.56-4.10)	0.66 (0.20-2.16)	1.08 (0.43-2.75)
Nucleic acid sampling team	0.41 (0.06-2.77)	3.11 (0.42-23.01)	2.71 (0.36-20.49)	2.78 (0.24-32.22)
Non-designated hospital	0.68 (0.38-1.23)	0.50 (0.26-0.95)*	0.80 (0.37-1.75)	0.83 (0.47-1.48)
History of chronic disease in family members (Reference: No)				
Yes	1.51 (0.91-2.50)	2.22 (1.30-3.78)**	1.35 (0.72-2.52)	2.03 (1.22-3.37)**

*P<0.05, **P<0.01, ***P<0.001 (univariate logistic regression).

Factors associated with self-reported anxiety symptoms in the Phase I survey included: age range of 30-50 years, history of chronic disease, nurse, administrative staff, work department is fever clinic or infectious disease department, and clinical laboratory, working less than 3 years, working 7-10 years, and average working hours per day less than 9 h since COVID-19 epidemic in Shanghai, workplace during COVID-19 epidemic in Shanghai was nucleic acid sampling team, and family member with a history of chronic disease (Table 1). In the Phase II survey, associated factors included: chronic disease, party affairs medical management department, working 7-10 years, average working hours since Shanghai has lifted lockdown less than 9 h, and between 9-10 h, working in the non-designated hospital, family member with a history of chronic disease (Table 2).

### Factors associated with stress and PTSD symptoms

Factors associated with self-reported stress symptoms in the Phase I survey included: history of chronic disease, nurse, administrative staff, logistics support department, working 7-10 years, and average working hours per day less than 9 h since the epidemic, and family member with a history of chronic disease (Table 1). In the Phase II survey, associated factors included: undergraduate educational level, chronic disease, fever clinic or infectious disease department, emergency or intensive care unit or hemodialysis room, clinical laboratory, working 7-10 years, and average working hours since Shanghai has lifted lockdown less than 9 h and between 9-10 h (Table 2).

Factors associated with self-reported PTSD symptoms in the Phase I survey included: educational level junior college or less, history of chronic disease, and average working hours per day since epidemic less than 9 h (Table 1). In the Phase II survey, associated factors included: chronic disease and family member with a history of chronic disease (Table 2).

### Factors associated with fatigue symptoms

The fatigue mean score was 121.23±45.77 in the Phase I survey and 144.73±44.14 in the Phase II survey. Factors associated with fatigue scores in the Phase I survey were gender, education level, occupation, department of work, hospital type, technical title, working years, average working hours per day since the epidemic, workplace and living places during the COVID-19 epidemic in Shanghai, and whether family members had a history of chronic diseases (P<0.05) (Supplementary Table S4). The Phase II survey revealed significant differences in fatigue levels between categories for all factors except for hospital type and marital status (Supplementary Table S5).

Furthermore, we conducted multiple linear regression analyses to explore the factors associated with fatigue levels of the medical staff in the Phase I survey (Supplementary Table S6). Individuals younger than 30 (P<0.001) and between 30 and 50 years of age (P<0.001), as well as those with chronic disease (P<0.001), were more likely to experience fatigue. Compared to other work positions, doctors, nurses, medical technicians, and administrative staff were positively correlated with fatigue scores (P<0.001). Moreover, there was a positive association between fatigue symptoms and medical staff working in the mobile cabin hospital (P<0.001), nucleic acid sampling team (P=0.002), and original hospital (P=0.015) compared to other workplaces. Medical workers in the department of emergency or intensive care unit or hemodialysis room (P<0.001) and other clinical departments (P=0.011) were also positively associated with fatigue scores. In contrast, staff in the logistics support department (P<0.001) had a negative relationship with fatigue scores. Working between 3 and 6 years (P<0.001), working 7-10 years (P<0.001), an average working time of 9-10 h per day during the COVID-19 outbreak in Shanghai (P=0.027), and individuals with higher scores on the IES-R scale (P<0.001) were positively correlated with the level of fatigue. It is worth noting that work in the logistics support department (β=-0.123, P<0.001), no technical title (β=-0.077, P=0.039), and higher DASS-21 anxiety scores (β=-0.170, P<0.001) were significantly associated to lower fatigue scores.

In the Phase II survey (Supplementary Table S7), factors positively associated with a higher level of fatigue included males (P=0.004), age 30-50 years (P=0.032), undergraduate (P=0.014), with chronic disease (P<0.001), doctors (P<0.001), nurses (P<0.001), medical technicians (P<0.001), administrative staff (P<0.001), working in fever clinic or infectious disease department (P<0.001), working in emergency or intensive care unit or hemodialysis room (P<0.001), working in the clinical laboratory (P<0.001), junior technical title (P=0.004), working years between 3 and 6 (P<0.001), working 7-10 years (P<0.001), working in the non-designated hospital (P<0.001), living with family (P=0.003), staying at quarantine hotel (P=0.007), family members had a chronic disease (P<0.001), higher DASS-21 depression scores (P=0.002), and higher IES-R scores (P<0.001). Apart from those three factors in the Phase I study, average working hours fewer than 9 per day (β=-0.463, P<0.001), between 9 and 10 h (β=-0.327, P<0.001), between 11 and 12 h (β=-0.197, P=0.006) were also significantly associated with lower fatigue scores in the Phase II survey.

## Discussion

### Main findings

This study aimed to evaluate the psychological symptoms of medical personnel following the COVID-19 outbreak in Shanghai and to explore the related influencing factors. More than 2,000 valid questionnaires were included in the Phase I survey whereas the Phase II survey, which was conducted four months later, had less valid follow-up responses, making longitudinal comparisons impractical. Nonetheless, from an overall perspective, the proportion of medical professionals experiencing depression, anxiety, and PTSD symptoms was generally higher in the Phase I survey. The situation of COVID-19 in Shanghai was severe, with thousands of asymptomatic infections and confirmed cases every day in Phase I (20). Moreover, despite the resumption of work and production in Shanghai, it appeared that a higher proportion of the medical staff was under stress and had an overall higher fatigue level than the general population. Medical staff developed psychological symptoms after long-term and high-intensity work during the COVID-19 epidemic in Shanghai. In Phase II, the medical staff resumed their regular duties as the Shanghai pandemic had been largely brought under control. Meanwhile, medical professionals had been under immense pressure due to concerns about the physical health of the elderly living at home and the learning and living conditions of children after the epidemic.

### Prevalence rate of anxiety and depression symptoms compared to previous studies

According to a meta-analysis that included research published before March 2021, during the COVID-19 pandemic, the prevalence of anxiety problems among Chinese health workers was 27%, while the prevalence of depression problems was 29%, with frontline workers exhibiting notably higher levels of both symptoms (21). For the COVID-19 outbreak in Shanghai, the comparatively high prevalence of anxiety and depression symptoms among medical staff may be attributed to the Omicron strain. The Omicron BA.2 and BA.2.2 mutant strains mainly prevalent in Shanghai were novel variants and prior treatment protocols were not fully applicable to this outbreak, making treatment more difficult. Furthermore, this mutant strain was more contagious (22), leading to a massive number of people contracting COVID-19. This, coupled with the overlap with the routine treatment of patients with chronic diseases, exceeded the capacity of available medical resources. The sudden COVID-19 outbreak in Shanghai put an overwhelming strain on its medical resources raising the risk of medical staff practice and causing great physical and psychological burden. Thus, the prevalence of depression and anxiety symptoms was higher than in previous investigations.

### Prevalence rate of stress and PTSD symptoms compared to previous studies

Our findings demonstrated that the distribution of stress prevalence among medical staff in Phase I and Phase II investigations was 20.35 and 28.23%, both lower than those reported in domestic and international studies at the beginning of the COVID-19 epidemic (23). Notably, stress prevalence was slightly higher in Phase II investigations compared to Phase I. Medical professionals are psychologically resilient and, to a certain extent, capable of modulating their mental state to cope with negative events (24). Consequently, medical staff with different levels of mental resilience may have reacted differently to this public health emergency, which potentially explains the decrease in anxiety prevalence. However, in both the Phase I and Phase II surveys, the medical staff was faced with considerable stress, in terms of worrying about their personal health as well as that of their family, friends, and colleagues and longer working hours due to crowded medical resources and staff shortages (25). Notably, the results of the Phase I survey indicated that administrative staff also reported higher levels of stress, whereas those working in the logistics department should be considered separately in the analysis of our study.

Although scale cut-offs may overestimate the prevalence of psychological symptoms (26), PTSD symptoms among medical personnel during the COVID-19 epidemic remain worthy of concern. Earlier studies conducted during the COVID-19 outbreak in various locations including Shanghai suggested a comparably high prevalence of PTSD symptoms among medical staff, which decreased over time (27). In addition, the medical staff with chronic illnesses was more likely to suffer from PTSD symptoms in the Phase I survey. Family members with chronic illnesses were also observed to be a risk factor for PTSD symptoms in the Phase II survey. The abrupt surge in workload during the sudden COVID-19 outbreak exposed medical staff to acute stressful events, leading to heightened negative emotions and mental fatigue. Long working hours and lack of sleep are two critical factors that contribute to mental fatigue (28). Our study demonstrated a general trend that longer average daily working hours were associated with higher levels of fatigue. In addition, medical professionals who suffered from PTSD and depression symptoms felt higher levels of fatigue.

In the specific context of the COVID-19 epidemic in Shanghai, healthcare workers were already experiencing significant stress due to their demanding schedules. Frontline clinical workers, in particular, may have been susceptible to compassion fatigue, as suggested by existing literature on the mental health of nurses during outbreaks (29). While our study did not directly measure compassion fatigue, it is a recognized psychological phenomenon among frontline medical staff who may excessively prioritize patient care during crises such as COVID-19. The Compassion Fatigue Model posits that without adequate coping resources, medical personnel can develop symptoms akin to PTSD symptoms (30).

### Factors correlated to psychological symptoms

Medical professionals themselves or their family members with chronic diseases were more prone to develop psychological problems. Potential explanations included the medical staff's concern that persons with chronic conditions were more vulnerable to COVID-19. Alternatively, the medical staff may be concerned that having COVID-19 along with chronic illnesses may have more severe repercussions. Prior studies indicated that those with chronic illnesses who are exposed to COVID-19 are more likely to experience negative effects (31). In addtion, due to the severity of the COVID-19 epidemic in Shanghai, medical institutions stopped providing several disease-curing treatments (32). Moreover, the prevention and treatment of COVID-19 required a significant amount of medical resources, making several routine hospital duties more difficult. Thus, medical professionals were concerned that it could be difficult for them or their families to receive healthcare for their chronic conditions during the COVID-19 outbreak.

Due to the high transmission capacity of the COVID-19 virus, clinical frontline staff members exhibited elevated anxiety and depression symptoms. Frontline healthcare staff with direct patient contact, especially those whose bodily fluids were utilized for PCR tests, reported increased levels of depression, anxiety, and PTSD symptoms (33). In our study, administrative staff who worked during and after the COVID-19 epidemic reported that they were responsible for temporary system setup, supply acquisition, distribution, and movement, patient transfer and treatment coordination, taking care of the families of frontline relief workers, preventing and isolating nosocomial infections, etc. They may be more susceptible to suffering from negative emotions from increased workload and obligations to patients and medical personnel. Although managerial support can aid other medical staff who are battling COVID-19 in maintaining their mental health (34), the more time and effort the administrative staff spend may increase their own occupational stress and exhaustion (35).

Compared to administrative staff, clinical medical staff exhibited significantly higher levels of stress, anxiety, depression, and PTSD symptoms. Their psychological barriers may be further exacerbated by prolonged close contact with confirmed patients, their inability to help infected patients, and their fear of spreading the virus to their families (36).

During such a public health emergency, medical workers with fewer working years may have difficulty sustaining mental wellness. Medical personnel with fewer working years seemed to be more at risk for depression and PTSD symptoms, possibly due to their lack of clinical knowledge and poor ability to adjust to the pandemic, according to a prior study (10) conducted in emergency departments during COVID-19. Previous exposure to epidemics such as SARS made medical personnel less afraid and gave them more drive to overcome it (37). Nevertheless, medical professionals with seven to ten years of experience were at risk of developing mental health problems. The majority of these medical professionals needed to care for their young children and support their aged parents. These medical professionals are under much more pressure due to their worries about how to ensure their parents' daily life, how to timely treat their parents' illnesses, and how to ensure their children's education while they are away (38).

Workers with shorter working hours each day experienced a better psychological state. Poor sleep quality, irregular dietary patterns, and long working hours may exacerbate mental health problems (39). Medical professionals with shorter working hours have less psychological stress, so they can get better rest and are more able to stay energized throughout the COVID-19 epidemic in Shanghai.

Regarding the workplace, medical professionals who worked in cabin hospitals and designated hospitals reported significantly lower levels of anxiety symptoms during the initial stage of this epidemic. Cabin hospitals and designated hospitals were constructed under the government's guidance for the COVID-19 pandemic. Medical staff that worked in a cabin hospital or designated hospital experienced less emotional exhaustion, according to research conducted during the same period (40). Compared to other types of hospitals, social support and a secure workplace are better in these two types of hospitals. Medical workers in non-designated facilities, which had less exposure to COVID-19 patients, exhibited much less depression after the full resumption of work and production in Shanghai. Thus, medical professionals may have experienced less stress, with fewer cases exhibiting psychological symptoms.

### Limitations

There were some limitations in our study. Firstly, despite our best attempts to ensure the quality of the questionnaire, its online format due to the limitations of the COVID-19 epidemic might hinder it from being as effective as a face-to-face survey. It is also hard to completely rule out the misreporting of self-reported data. Additionally, a longitudinal comparison was challenging because the samples from the two stages were not precisely the same. Furthermore, this study did not identify the specific traumatic events via the DSM-IV scale. Therefore, the extrapolation of results ought to be undertaken cautiously. Moreover, the small sample size of some subgroups such as medical staff working in primary and secondary hospitals were not representative of the overall situation. Hence a larger sample size of these subgroups is necessary to validate the results.

## Conclusions

The current study indicated that medical staff in Shanghai suffered from varying degrees of depression, anxiety, stress, PTSD symptoms, and fatigue symptoms. Longer average workdays and chronic illnesses were associated with a higher risk of psychological problems in medical workers. In addition, administrative staff and clinical frontline healthcare workers such as doctors and nurses were far more likely to experience mental health issues. To support future public health emergencies, suitable and targeted interventions for healthcare workers should be implemented, considering the various stages of the epidemic and the various characteristics of healthcare professionals.

## Supplementary Material

Click to view [pdf].
