# Supplementary material for: Factors associated with depression, anxiety, stress, PTSD, and fatigue of medical staff during the COVID-19 pandemic in Shanghai: a two-phase cross-sectional study
Source: Braz J Med Biol Res. 2025 Mar 3;58:e13943. doi: 10.1590/1414-431X2024e13943 (PMC11884776; doi:10.1590/1414-431X2024e13943)
Supplement: Supplementary file 1 [file 1414-431X-bjmbr-58-e13943-suppl.pdf]

Table S1. Sociodemographic information of medical staff in Shanghai during the COVID-19 epidemic.

Characteristics	Phase I survey	Phase II survey
	Cases (%)	Cases (%)
Gender		
Male	602 (27.5%)	528 (51.2%)
Female	1590 (72.5%)	503 (48.8%)
Age		
<30	512 (23.4%)	120 (11.6%)
30–50	1509 (68.8%)	816 (79.1%)
>50	171 (7.8%)	95 (9.2%)
Educational level		
Junior college or less	689 (31.4%)	269 (26.1%)
Undergraduate	1078 (49.2%)	693 (67.2%)
Postgraduate or more	425 (19.4%)	69 (6.7%)
Marital status		
Married	1565 (71.4%)	835 (81.0%)
Others	627 (28.6%)	196 (19.0%)
History of chronic disease		
Yes	407 (18.6%)	723 (70.1%)
No	1785 (81.4%)	308 (29.9%)
Occupation		
Doctor	425 (19.4%)	382 (37.1%)
Nurse	1139 (52.0%)	490 (47.5%)
Medical technicians	249 (11.4%)	89 (8.6%)
Administrative staff	71 (3.2%)	39 (3.8%)
Others	308 (14.1%)	31 (3.0%)
Department of work		
Fever clinic/Infectious disease department	48 (2.2%)	226 (21.9%)
Emergency/Intensive care unit/Hemodialysis room	141 (6.4%)	260 (25.2%)
Clinical laboratory	109 (5.0%)	234 (22.7%)
Other clinical departments	1292 (58.9%)	158 (15.3%)
Party affairs medical management department	28 (1.3%)	17 (1.6%)
Logistics support department	162 (7.4%)	24 (2.3%)
Others	412 (18.8%)	112 (10.9%)
Hospital type		
Primary hospital	19 (0.9%)	1 (0.1%)
Secondary hospital	31 (1.4%)	8 (0.8%)
Tertiary hospital	2142 (97.7%)	1022 (99.1%)
Technical title		
No	503 (22.9%)	47 (4.6%)
Junior	881 (40.2%)	810 (78.6%)
Intermediate	638 (29.1%)	141 (13.7%)
Senior	170 (7.7%)	33 (3.2%)
Working years (years)		
<3	404 (18.4%)	74 (7.2%)
3–6	500 (22.8%)	407 (39.5%)
7–10	376 (17.2%)	299 (29.0%)
>10	912 (41.6%)	251 (24.3%)
Average working hours since epidemic (hours/day)		
<9	1190 (54.3%)	241 (23.4%)
9–10	546 (24.9%)	301 (29.2%)
11–12	266 (12.1%)	440 (42.7%)
>12	190 (8.7%)	49 (4.8%)
Present workplace		
Mobile cabin hospital	524 (23.9%)	40 (3.9%)
Designated hospital	275 (12.5%)	7 (0.7%)
Nucleic acid sampling team	39 (1.8%)	882 (85.5%)
Stayed at the hospital	829 (37.8%)	102 (9.9%)
Others	525 (24.0%)	
Present living place		
Living in a closed loop in workplace	954 (43.5%)	33 (3.2%)
Living alone	283 (12.9%)	962 (93.3%)
Living with family	802 (36.6%)	31 (3.0%)
Others	153 (7.0%)	5 (0.5%)
History of chronic disease in family members		
Yes	1033 (47.1%)	792 (76.8%)
No	1159 (52.9%)	239 (23.2%)

Table S2. Prevalence of depression, anxiety, stress, and post-traumatic stress disorder (PTSD) among medical staff in the Phase I survey.

Characteristics	Depression (n=997) n (%)	χ^2	P	Anxiety (n=919) n (%)	χ^2	P	Stress (n=446) n (%)	χ^2	P	PTSD (n=1656) n (%)	χ^2	P
Gender												
Male	263 (43.7%)	1.079	0.299	243 (40.4%)	0.829	0.362	122 (20.3%)	0.003	0.954	432 (71.8%)	6.442	**0.011**
Female	734 (46.2%)			676 (42.5%)			324 (20.4%)			1224 (77.0%)		
Age												
<30	234 (45.7%)	12.334	**0.002**	222 (43.4%)	15.896	**<0.001**	105 (20.5%)	5.518	0.063	366 (71.5%)	14.224	**0.001**
30–50	707 (46.9%)			650 (43.1%)			318 (21.1%)			1174 (77.8%)		
>50	56 (32.7%)			47 (27.5%)			23 (13.5%)			116 (67.8%)		
Educational level												
Junior college or less	291 (42.2%)	6.736	**0.034**	275 (39.9%)	31.565	**<0.001**	119 (17.3%)	6.468	**0.039**	485 (70.4%)	16.277	**<0.001**
Undergraduate	520 (48.2%)			509 (47.2%)			240 (22.3%)			850 (78.8%)		
Postgraduate or more	186 (43.8%)			135 (31.8%)			87 (20.5%)			321 (75.5%)		
Marital status												
Married	691 (44.2%)	4.012	0.135	645 (41.2%)	1.137	0.566	309 (19.7%)	1.225	0.542	1187 (75.8%)	0.516	0.773
Others	306 (48.8%)			274 (43.7%)			137 (21.9%)			469 (74.8%)		
History of chronic disease												
Yes	240 (59.0%)	36.651	**<0.001**	240 (59.0%)	59.624	**<0.001**	111 (27.3%)	14.793	**<0.001**	339 (83.3%)	16.229	**<0.001**
No	757 (42.4%)			679 (38.0%)			335 (18.8%)			1317 (73.8%)		
Occupation												
Doctor	206 (48.5%)	90.395	**<0.001**	170 (40.0%)	103.125	**<0.001**	90 (21.2%)	40.282	**<0.001**	326 (76.7%)	35.403	**<0.001**
Nurse	592 (52.0%)			577 (50.7%)			268 (23.5%)			904 (79.4%)		
Medical technicians	91 (36.5%)			74 (29.7%)			41 (16.5%)			180 (72.3%)		
Administrative staff	36 (50.7%)			31 (43.7%)			21 (29.6%)			50 (70.4%)		
Others	72 (23.4%)			67 (21.8%)			26 (8.4%)			196 (63.6%)		
Department of work												
Fever clinic/Infectious disease department	18 (37.5%)	63.325	**<0.001**	15 (31.3%)	63.774	**<0.001**	9 (18.8%)	25.661	**<0.001**	33 (68.8%)	28.349	**<0.001**
Emergency/Intensive care unit/Hemo-dialysis room	83 (58.9%)			83 (58.9%)			37 (26.2%)			117 (83.0%)		
Clinical laboratory	53 (48.6%)			50 (45.9%)			24 (22.0%)			86 (78.9%)		
Other clinical departments	625 (48.4%)			586 (45.4%)			278 (21.5%)			1005 (77.8%)		
Party affairs medical management department	13 (46.4%)			11 (39.3%)			9 (32.1%)			21 (75.0%)		
Logistics support department	31 (19.1%)			36 (22.2%)			11 (6.8%)			102 (63.0%)		
Others	174 (42.2%)			138 (33.5%)			78 (18.9%)			292 (70.9%)		
Hospital type												
Primary hospital	9 (47.4%)	4.630	0.099	9 (47.4%)	3.618	0.164	7 (36.8%)	3.328	0.189	16 (84.2%)	1.796	0.407
Secondary hospital	20 (64.5%)			18 (58.1%)			7 (22.6%)			21 (67.7%)		
Tertiary hospital	968 (45.2%)			892 (41.6%)			432 (20.2%)			1619 (75.6%)		
Technical title												
No	178 (35.4%)	28.855	**<0.001**	157 (31.2%)	40.055	**<0.001**	74 (14.7%)	15.091	**0.005**	332 (66.0%)	40.925	**<0.001**
Junior	433 (49.1%)			412 (46.8%)			192 (21.8%)			692 (78.5%)		
Intermediate	309 (48.4%)			286 (44.8%)			142 (22.3%)			503 (78.8%)		
Senior	93 (54.7%)			64 (37.6%)			38 (22.4%)			129 (75.9%)		
Working years												
<3	173 (42.8%)	17.967	**<0.001**	156 (38.6%)	14.283	**0.003**	67 (16.6%)	12.917	**0.005**	273 (67.6%)	22.985	**<0.001**
3–6	239 (47.8%)			233 (46.6%)			111 (22.2%)			368 (73.6%)		
7–10	203 (54.0%)			177 (47.1%)			97 (25.8%)			302 (80.3%)		
>10	382 (41.9%)			353 (38.7%)			171 (18.8%)			713 (78.2%)		

Average working hours since epidemic (hours/day)												
<9	497 (41.8%)	15.165	**0.002**	408 (34.3%)	66.456	**<0.001**	189 (15.9%)	33.915	**<0.001**	853 (71.7%)	22.925	**<0.001**
9–10	277 (50.7%)			292 (53.5%)			143 (26.2%)			443 (81.1%)		
11–12	133 (50.0%)			133 (50.0%)			72 (27.1%)			205 (77.1%)		
>12	90 (47.4%)			86 (45.3%)			42 (22.1%)			155 (81.6%)		
Present workplace												
Mobile cabin hospital	250 (47.7%)	22.280	**<0.001**	265 (50.6%)	58.614	**<0.001**	123 (23.5%)	20.378	**<0.001**	401 (76.5%)	3.926	0.416
Designated hospital	104 (37.8%)			114 (41.5%)			53 (19.3%)			206 (74.9%)		
Nucleic acid sampling team	28 (71.8%)			26 (66.7%)			12 (30.8%)			32 (82.1%)		
Stayed at the hospital	394 (47.5%)			358 (43.2%)			184 (22.2%)			635 (76.6%)		
Others	221 (42.1%)			156 (29.7%)			74 (14.1%)			382 (72.8%)		
Present Living place												
Living in a closed loop in workplace	423 (44.3%)	7.634	0.054	416 (43.6%)	17.871	**<0.001**	211 (22.1%)	17.312	**0.001**	723 (75.8%)	0.519	0.915
Living alone	144 (50.9%)			144 (50.9%)			76 (26.9%)			217 (76.7%)		
Living with family	372 (46.4%)			300 (37.4%)			133 (16.6%)			603 (75.2%)		
Others	58 (37.9%)			59 (38.6%)			26 (17.0%)			113 (73.9%)		
History of chronic disease in family members												
Yes	541 (52.4%)	37.384	**<0.001**	516 (50.0%)	51.694	**<0.001**	257 (24.9%)	24.762	**<0.001**	821 (79.5%)	16.333	**<0.001**
No	456 (39.3%)			403 (34.8%)			189 (16.3%)			835 (72.0%)		

Table S3. Prevalence of depression, anxiety, stress, and post-traumatic stress disorder (PTSD) among medical staff in the Phase II survey.

Characteristics	Depression (n=204) n (%)	χ^2	P	Anxiety (n=221) n (%)	χ^2	P	Stress (n=291) n (%)	χ^2	P	PTSD (n=126) n (%)	χ^2	P
Gender												
Male	51 (9.7%)	69.938	**<0.001**	56 (10.6%)	75.366	**<0.001**	73 (13.8%)	110.764	**<0.001**	30 (5.7%)	43.141	**<0.001**
Female	153 (30.4%)			165 (32.8%)			218 (43.3%)			96 (19.1%)		
Age												
<30	34 (28.3%)	30.972	**<0.001**	34 (28.3%)	30.370	**<0.001**	45 (37.5%)	34.736	**<0.001**	24 (20.0%)	12.096	**0.002**
30–50	134 (16.4%)			148 (18.1%)			198 (24.3%)			85 (10.4%)		
>50	36 (37.9%)			39 (41.1%)			48 (50.5%)			17 (17.9%)		
Educational level												
Junior college or less	50 (18.6%)	12.601	**0.002**	55 (20.4%)	30.763	**<0.001**	73 (27.1%)	26.440	**<0.001**	35 (13.0%)	5.140	0.077
Undergraduate	129 (18.6%)			133 (19.2%)			180 (26.0%)			77 (11.1%)		
Postgraduate or more	25 (36.2%)			33 (47.8%)			38 (55.1%)			14 (20.3%)		
Marital status												
Married	149 (17.8%)	10.440	**0.001**	161 (19.3%)	12.102	**0.001**	213 (25.5%)	15.994	**<0.001**	89 (10.7%)	9.996	**0.002**
Others	55 (28.1%)			60 (30.6%)			78 (39.8%)			37 (18.9%)		
History of chronic disease												
Yes	34 (4.7%)	346.945	**<0.001**	33 (4.6%)	409.051	**<0.001**	60 (8.3%)	474.341	**<0.001**	14 (1.9%)	238.635	**<0.001**
No	170 (55.2%)			188 (61.0%)			231 (75.0%)			112 (36.4%)		
Occupation												
Doctor	18 (4.7%)	221.291	**<0.001**	22 (5.8%)	232.862	**<0.001**	24 (6.3%)	296.423	**<0.001**	10 (2.6%)	109.601	**<0.001**
Nurse	94 (19.2%)			99 (20.2%)			142 (29.0%)			64 (13.1%)		
Medical technicians	40 (44.9%)			47 (52.8%)			63 (70.8%)			23 (25.8%)		
Administrative staff	29 (74.4%)			27 (69.2%)			34 (87.2%)			13 (33.3%)		
Others	23 (74.2%)			26 (83.9%)			28 (90.3%)			16 (51.6%)		
Department of work												
Fever clinic/Infectious disease department	9 (4.0%)	434.513	**<0.001**	9 (4.0%)	486.583	**<0.001**	15 (6.6%)	650.383	**0.001**	7 (3.1%)	214.404	**<0.001**
Emergency/Intensive care unit/Hemo-dialysis unit/Hemo-dialysis room	12 (4.6%)			10 (3.8%)			16 (6.2%)			9 (3.5%)		
Clinical laboratory	4 (1.7%)			4 (1.7%)			4 (1.7%)			2 (0.9%)		
Other clinical departments	87 (55.1%)			99 (62.7%)			126 (79.7%)			52 (32.9%)		
Party affairs medical management department	16 (94.1%)			15 (88.2%)			17 (100.0%)			6 (35.3%)		
Logistics support department	21 (87.5%)			20 (83.3%)			23 (95.8%)			11 (45.8%)		
Others	55 (49.1%)			64 (57.1%)			90 (80.4%)			39 (34.8%)		
Hospital type												
Primary hospital	1 (100%)	6.031	**0.049**	1 (100.0%)	5.583	0.054	1 (100.0%)	5.700	0.058	0 (0.0%)	1.264	0.532
Secondary hospital	0 (0.0%)			0 (0.0%)			0 (0.0%)			0 (0.0%)		
Tertiary hospital	203 (19.9%)			220 (21.5%)			290 (28.4%)			126 (12.3%)		
Technical title												
No	37 (78.7%)	236.040	**<0.001**	39 (83.0%)	250.409	**<0.001**	43 (91.5%)	274.976	**<0.001**	26 (55.3%)	131.295	**<0.001**
Junior	84 (10.4%)			93 (11.5%)			133 (16.4%)			56 (6.9%)		
Intermediate	64 (45.4%)			67 (47.5%)			89 (63.1%)			34 (24.1%)		
Senior	19 (57.6%)			22 (66.7%)			26 (78.8%)			10 (30.3%)		
Working years												
<3	29 (39.2%)	283.271	**<0.001**	33 (44.6%)	338.303	**<0.001**	34 (45.9%)	453.322	**<0.001**	20 (27.0%)	148.263	**<0.001**
3–6	21 (5.2%)			19 (4.7%)			33 (8.1%)			16 (3.9%)		
7–10	20 (6.7%)			21 (7.0%)			28 (9.4%)			11 (3.7%)		
>10	134 (53.4%)			148 (59.0%)			196 (78.1%)			79 (31.5%)		

Average working hours since Shanghai is fully liberated (hours/day)												
<9	135 (56.0%)	286.402	**<0.001**	151 (62.7%)	343.206	**<0.001**	188 (78.0%)	431.844	**<0.001**	81 (33.6%)	149.102	**<0.001**
9–10	54 (17.9%)			55 (18.3%)			81 (26.9%)			34 (11.3%)		
11–12	12 (2.7%)			12 (2.7%)			17 (3.9%)			8 (1.8%)		
>12	3 (6.1%)			3 (6.1%)			5 (10.2%)			3 (6.1%)		
Present workplace												
Designated hospital	16 (40.0%)	96.507	**<0.001**	17 (42.5%)	100.439	**<0.001**	30 (75.0%)	196.264	**<0.001**	11 (27.5%)	59.275	**<0.001**
Nucleic acid sampling team	4 (57.1%)			2 (28.6%)			4 (57.1%)			1 (14.3%)		
Non-designated hospital	131 (14.9%)			144 (16.3%)			178 (20.2%)			80 (9.1%)		
Others	53 (52.0%)			58 (56.9%)			79 (77.5%)			34 (33.3%)		
Present living place												
Living alone	18 (54.5%)	44.646	**<0.001**	18 (54.5%)	40.444	**<0.001**	23 (69.7%)	47.114	**<0.001**	9 (27.3%)	22.048	**<0.001**
Living with family	182 (18.9%)			199 (20.7%)			264 (27.4%)			114 (11.9%)		
Stayed at quarantine hotel	0 (0.0%)			0 (0.0%)			0 (0.0%)			0 (0.0%)		
Others	4 (80.0%)			4 (80.0%)			4 (80.0%)			3 (60.0%)		
History of chronic disease in family members												
Yes	82 (10.4%)	191.547	**<0.001**	82 (10.4%)	249.149	**<0.001**	127 (16.0%)	250.589	**<0.001**	44 (5.6%)	141.502	**<0.001**
No	122 (51.0%)			139 (58.2%)			164 (68.6%)			82 (34.3%)		

Table S4. Mean differences of fatigue scores by socio-demographic variables among medical staff in the Phase I survey.

Factors	N	FAI scores (χ±s)	F/t	P
Gender				
Male	602	106.85 ± 46.126	3.772	**<0.001**
Female	1590	126.67 ± 44.460		
Age				
<30	512	120.63 ± 46.347	0.611	0.543
30–50	1509	121.81 ± 45.408		
>50	171	117.92 ± 47.381		
Educational level				
Junior college or less	689	112.76 ± 49.440	17.427	**<0.001**
Undergraduate	1078	125.14 ± 44.262		
Postgraduate or more	425	125.02 ± 41.423		
Marital status				
Married	1565	120.30 ± 45.865	0.336	0.135
Others	627	123.54 ± 45.506		
History of chronic disease				
Yes	407	123.08 ± 46.048	0.258	0.365
No	1785	120.80 ± 45.716		
Occupation				
Doctor	425	112.25 ± 44.928	13.437	**<0.001**
Nurse	1139	126.49 ± 46.675		
Medical technicians	249	124.92 ± 39.101		
Administrative staff	71	126.42 ± 46.875		
Others	308	109.97 ± 44.682		
Department of work				
Fever clinic/Infectious disease department	48	125.21 ± 50.221	8.110	**<0.001**
Emergency/Intensive care unit/Hemodialysis room	141	138.83 ± 41.811		
Clinical laboratory	109	125.46 ± 43.116		
Other clinical departments	1292	119.62 ± 46.239		
Party affairs medical management department	28	122.50 ± 49.120		
Logistics support department	162	104.72 ± 48.241		
Others	412	125.07 ± 42.060		
Hospital type				
Primary hospital	19	122.11 ± 54.843	6.034	**0.002**
Secondary hospital	31	92.94 ± 50.569		
Tertiary hospital	2142	121.63 ± 45.514		
Technical title				
No	503	109.26 ± 47.181	24.042	**<0.001**
Junior	881	129.65 ± 44.720		
Intermediate	638	121.25 ± 43.619		
Senior	170	112.86 ± 45.850		
Working years				
<3	404	108.73 ± 48.687	30.980	**<0.001**
3–6	500	114.35 ± 46.815		
7–10	376	118.68 ± 46.228		
>10	912	131.58 ± 41.298		
Average working hours since epidemic (hours/day)				
<9	1190	122.00 ± 44.188	1.243	0.292
9–10	546	118.52 ± 48.641		
11–12	266	120.50 ± 48.269		
>12	190	125.17 ± 43.329		
Present workplace				
Cabin hospital	524	102.27 ± 46.983	45.091	**<0.001**
Designated hospital	275	117.93 ± 45.345		
Nucleic acid sampling team	39	98.13 ± 47.973		
Stayed at the hospital	829	134.00 ± 42.894		
Others	525	123.41 ± 41.732		

Present living place				
Living in a closed loop in workplace	954	125.43 ± 46.597	8.338	**<0.001**
Living alone	283	110.24 ± 46.568		
Living with family	802	120.38 ± 43.754		
Others	153	119.79 ± 46.236		
History of chronic disease in family members				
Yes	1033	129.39 ± 44.338	2.334	**<0.001**
No	1159	113.95 ± 45.830		

FAI: Fatigue Assessment Instrument. Student's *t*-test.

Table S5. Mean differences of fatigue scores by socio-demographic variables among medical staff in the Phase II survey.

Factors	N	FAI scores (χ±s)	F/t	P
Gender				
Male	528	148.59 ± 42.918	8.337	**0.004**
Female	503	140.68 ± 45.077		
Age				
<30	120	138.00 ± 45.784	3.899	**0.021**
30–50	816	146.69 ± 43.468		
>50	95	136.43 ± 46.400		
Educational level				
Junior college or less	269	138.86 ± 45.34	6.303	**0.002**
Undergraduate	693	148.04 ± 43.24		
Postgraduate or more	69	134.35 ± 44.98		
Marital status				
Married	835	144.65 ± 44.25	0.014	0.905
Others	196	145.07 ± 43.79		
History of chronic disease				
Yes	723	153.02 ± 40.06	93.051	**<0.001**
No	308	125.27 ± 47.13		
Occupation				
Doctor	382	163.77 ± 36.52	43.060	**<0.001**
Nurse	490	136.61 ± 41.6		
Medical technicians	89	135.87 ± 48.59		
Administrative staff	39	124.54 ± 42.08		
Others	31	89.42 ± 54.45		
Department of work				
Fever clinic/Infectious disease department	226	141.07 ± 43.63	25.417	**<0.001**
Emergency/Intensive care unit/Hemodialysis room	260	149.88 ± 38.51		
Clinical laboratory	234	167.23 ± 34.37		
Other clinical departments	158	132.78 ± 45.3		
Party affairs medical management department	17	122.41 ± 42.18		
Logistics support department	24	100.17 ± 51.76		
Others	112	122.96 ± 47.17		
Hospital type				
Primary hospital	1	116.00 ± 00.00	0.271	0.763
Secondary hospital	8	150.13 ± 46.54		
Tertiary hospital	1022	144.72 ± 44.16		
Technical title				
No	47	98.96 ± 50.69	24.359	**<0.001**
Junior	810	149.36 ± 41.52		
Intermediate	141	137.47 ± 47.11		
Senior	33	127.30 ± 39.55		
Working years				
<3	74	135.11 ± 52.79	13.571	**<0.001**
3–6	407	149.82 ± 41.86		
7–10	299	151.49 ± 40.92		
>10	251	131.27 ± 45.44		
Average working hours since epidemic (hours/day)				
<9	241	124.53 ± 45.64	35.638	**<0.001**
9–10	301	141.03 ± 43.65		
11–12	440	155.21 ± 39.56		
>12	49	172.76 ± 35.12		
Present workplace				
Designated hospital	40	140.65 ± 42.62	4.743	**0.003**
Nucleic acid sampling team	7	138 ± 41.31		
Stayed at the hospital	882	146.71 ± 43.45		
Others	102	129.72 ± 48.25		

Present living place				
Living in a closed loop in workplace	33	120.3 ± 46.89	6.496	**<0.001**
Living alone	962	145.87 ± 43.92		
Living with family	31	144.71 ± 34.73		
Others	5	87.4 ± 48.44		
History of chronic disease in family members				
Yes	792	151.15 ± 41.37	77.565	**<0.001**
No	239	123.47 ± 46.4		

FAI: Fatigue Assessment Instrument. Student's *t*-test.

Table S6. Multiple linear regression for fatigue of medical staff in the Phase I survey.

Variables	B	Standard Error	β	t	P Value
(Constant)	9.278	0.213		43.470	**<0.001**
Gender (Reference: Female)					
Male	–0.291	0.407	–0.015	–0.715	0.475
(Constant)	6.596	0.648		10.173	**<0.001**
Age (Reference: >50)					
<30	2.900	0.749	0.144	3.872	**<0.001**
30–50	2.795	0.684	0.152	4.085	**<0.001**
(Constant)	8.908	0.412		21.602	**<0.001**
Educational level (Reference: Postgraduate or more)					
Junior college or less	–0.241	0.524	–0.013	–0.459	0.646
Undergraduate	0.743	0.487	0.044	1.526	0.127
(Constant)	9.668	0.340		28.460	**<0.001**
Marital status (Reference: Others)					
Married	–0.659	0.402	–0.035	–1.638	0.101
(Constant)	8.605	0.199		43.176	**<0.001**
Chronic disease (Reference: No)					
Yes	3.193	0.463	0.146	6.904	**<0.001**
(Constant)	5.201	0.474		10.976	**<0.001**
Occupation (Reference: Others)					
Doctor	3.980	0.622	0.185	6.395	**<0.001**
Nurse	5.329	0.534	0.313	9.977	**<0.001**
Medical technicians	2.686	0.709	0.100	3.790	**<0.001**
Administrative staff	4.658	1.095	0.097	4.254	**<0.001**
(Constant)	8.544	0.413		20.673	**<0.001**
Department of work (Reference: others)					
Fever clinic/Infectious disease department	–0.210	1.279	–0.004	–0.164	0.869
Emergency/Intensive care unit/Hemodialysis room	2.960	0.818	0.085	3.616	**<0.001**
Clinical laboratory	0.594	0.904	0.015	0.657	0.511
Other clinical departments	1.209	0.475	0.070	2.547	**0.011**
Party affairs medical management department	1.742	1.638	0.023	1.063	0.288
Logistics support department	–4.000	0.778	–0.123	–5.142	**<0.001**
(Constant)	9.192	0.184		49.975	**<0.001**
Hospital type (Reference: Tertiary hospital)					
Primary hospital	–0.456	1.962	–0.005	–0.232	0.816
Secondary hospital	0.679	1.540	0.009	0.441	0.660
(Constant)	9.035	0.649		13.927	**<0.001**
Technical title (Reference: Senior)					
No	–1.552	0.750	–0.077	–2.068	**0.039**
Junior	0.960	0.709	0.055	1.355	0.176
Intermediate	0.457	0.730	0.024	0.626	0.532
(Constant)	8.509	0.280		30.353	**<0.001**
Working years (Reference: >10)					
<3	–0.182	0.506	–0.008	–0.360	0.719
3–6	1.715	0.471	0.085	3.641	**<0.001**
7–10	1.933	0.519	0.086	3.725	**<0.001**
(Constant)	9.211	0.613		15.024	**<0.001**
Average working hours since epidemic (Reference: >12)					
<9	–0.890	0.660	–0.052	–1.347	0.178
9–10	1.577	0.712	0.080	2.216	**0.027**
11–12	0.639	0.803	0.025	0.796	0.426
(Constant)	8.217	0.370		22.218	**<0.001**
Present workplace (Reference: Others)					
Mobile cabin hospital	1.894	0.523	0.095	3.619	**<0.001**
Designated hospital	0.117	0.631	0.005	0.186	0.852
Nucleic acid sampling team	4.347	1.406	0.068	3.091	**0.002**
Stayed at the hospital	1.153	0.473	0.066	2.440	**0.015**

(Constant)	8.131	0.687		11.832	<0.001
Present living place (Reference: Others)					
Living in a closed loop in workplace	1.131	0.740	0.066	1.528	0.127
Living alone	2.173	0.853	0.086	2.548	0.011
Living with family	0.804	0.750	0.046	1.073	0.283
(Constant)	8.033	0.247		32.473	<0.001
History of chronic disease in family members (Reference: No)					
Yes	2.473	0.360	0.145	6.862	<0.001
(Constant)	84.029	1.446		58.105	**<0.001**
Values					
DASS-21 Depression	0.450	0.173	0.084	2.600	0.009
DASS-21 Anxiety	–0.968	0.201	–0.170	–4.806	**<0.001**
DASS-21 Stress	0.695	0.201	0.134	3.455	0.001
IES-R	1.707	0.089	0.509	19.166	**<0.001**

B: unstandardized coefficients; β: standardized coefficients. β=0.1 is considered small, β=0.3 is considered medium, and β=0.5 is considered large according to the guidelines by Cohen. DASS-21: Depression Anxiety Stress Scale-21; IES-R: Impact of Event Scale-Revised.

Table S7. Multiple linear regression for fatigue of medical staff in the Phase II survey.

Variables	B	Standard Error	β	t	P Value
(Constant)	140.680	1.961		71.733	**<0.001**
Gender (Reference: Female)					
Male	7.913	2.740	0.090	2.887	**0.004**
(Constant)	136.432	4.516		30.210	**<0.001**
Age (Reference: >50)					
<30	1.568	6.045	0.011	0.259	0.795
30–50	10.257	4.772	0.094	2.150	**0.032**
(Constant)	134.348	5.287		25.412	**<0.001**
Educational level (Reference: Postgraduate or more)					
Junior college or less	4.515	5.926	0.045	0.762	0.446
Undergraduate	13.697	5.544	0.146	2.471	**0.014**
(Constant)	145.071	3.154		45.990	**<0.001**
Marital status (Reference: Others)					
Married	–0.419	3.505	–0.004	–0.119	0.905
(Constant)	125.266	2.410		51.982	**<0.001**
Chronic disease (Reference: No)					
Yes	27.759	2.878	0.288	9.646	**<0.001**
(Constant)	89.419	7.350		12.165	**<0.001**
Occupation (Reference: Others)					
Doctor	74.348	7.643	0.814	9.728	**<0.001**
Nurse	47.191	7.579	0.534	6.226	**<0.001**
Medical technicians	46.446	8.535	0.296	5.442	**<0.001**
Administrative staff	35.119	9.847	0.152	3.566	**<0.001**
(Constant)	122.964	3.903		31.508	**<0.001**
Department of work (Reference: others)					
Fever clinic/Infectious disease department	18.102	4.773	0.170	3.793	**<0.001**
Emergency/Intensive Care Unit/Hemodialysis Room	26.916	4.668	0.265	5.766	**<0.001**
Clinical laboratory	44.266	4.746	0.420	9.328	**<0.001**
Other clinical departments	9.821	5.102	0.080	1.925	0.055
Party affairs medical management department	–0.553	10.750	–0.002	–0.051	0.959
Logistics support department	–22.798	9.290	–0.078	–2.454	**0.014**
(Constant)	144.718	1.382		104.737	**<0.001**
Hospital type (Reference: Tertiary hospital)					
Primary hospital	–28.718	44.194	–0.020	–0.650	0.516
Secondary hospital	5.407	15.678	0.011	0.345	0.730
(Constant)	127.303	7.435		17.122	**<0.001**
Technical title (Reference: Senior)					
No	–28.346	9.700	–0.134	–2.922	**0.004**
Junior	22.060	7.585	0.205	2.908	**0.004**
Intermediate	10.165	8.260	0.079	1.231	0.219
(Constant)	131.271	2.736		47.970	**<0.001**
Working years (Reference: >10)					
<3	3.837	5.735	0.022	0.669	0.504
3–6	18.547	3.479	0.205	5.331	**<0.001**
7–10	20.221	3.711	0.208	5.448	**<0.001**
(Constant)	172.755	6.010		28.745	**<0.001**
Average working hours since Shanghai is fully liberated (Reference: >12)					
<9	–48.224	6.593	–0.463	–7.315	**<0.001**
9–10	–31.722	6.481	–0.327	–4.895	**<0.001**
11–12	–17.548	6.336	–0.197	–2.770	**0.006**
(Constant)	129.716	4.347		29.841	**<0.001**
Present workplace (Reference: Others)					
Designated hospital	10.934	8.190	0.048	1.335	0.182
Nucleic acid sampling team	8.284	17.153	0.015	0.483	0.629
Non-designated hospital	16.992	4.591	0.135	3.701	**<0.001**
(Constant)	87.400	19.584		4.463	**<0.001**
Present living place (Reference: Others)					
Living alone	32.903	21.016	0.131	1.566	0.118

Living with family	58.469	19.635	0.331	2.978	**0.003**
Stayed at quarantine hotel	57.310	21.105	0.222	2.716	**0.007**
(Constant)	123.469	2.755		44.821	**<0.001**
History of chronic disease in family members (Reference: No)					
Yes	27.680	3.143	0.265	8.807	**<0.001**
(Constant)	72.690	8.181		8.885	**<0.001**
Values					
DASS-21 Depression	0.769	0.251	0.246	3.066	**0.002**
DASS-21 Anxiety	–0.828	0.287	–0.282	–2.886	**0.004**
DASS-21 Stress	0.374	0.284	0.121	1.315	0.189
IES-R	1.333	0.145	0.560	9.203	**<0.001**

B: unstandardized coefficients; β: standardized coefficients. β=0.1 small, β=0.3 medium, and β=0.5 large according to the guidelines by Cohen. DASS-21: Depression Anxiety Stress Scale-21; IES-R: Impact of Event Scale-Revised.
